# Determining the critical size of a rabbit rib segmental bone defect model

**DOI:** 10.1093/rb/rbw028

**Published:** 2016-09-20

**Authors:** Fengzhen Liu, Kun Chen, Lei Hou, Keyi Li, Dawei Wang, Bin Zhang, Xiumei Wang

**Affiliations:** ^1^Liaocheng People’s Hospital, Medical College of Liaocheng University, Liaocheng 252000, P. R. China;; ^2^Tsinghua University, Beijing 100084, P. R. China

**Keywords:** animal test, bone, critical size defect, rib defect, periosteum

## Abstract

In order to establish and standardize the rabbit rib segmental bone defect model, it is of vital importance to determine rabbit rib critical size defect (CSD). According to the general time needed for spontaneous long-bone regeneration, three-month observation period was set to determine the CSD. The rabbit rib segmental bone defects with different sizes from 1 to 5 cm with or without periosteum were performed in the eighth rib of 4-month-old male New Zealand rabbits and underwent X-ray examinations at the 4th, 8th and 12th weeks postoperatively. The gross and histological examinations at postoperative week 12 were evaluated, which showed that the critical sizes in the rabbit rib models with and without periosteum were 5 and 2 cm, respectively. This study provides prerequisite data for establishing rabbit rib CSD model and evaluating bone materials using this model.

## Introduction

Autologous rib is one of the most widely used bone autografts for repairing large segmental bone defects in clinic. For example, oral and maxillofacial surgeons commonly use autologous rib to repair the mandibular defects [1] and neurosurgeons also use autologous rib to repair a large and complex skull defects [2, 3]. Traditionally, the defective ribs are not repaired after being harvested, which actually will lead to a lot of adverse influences, such as chest wall deformities, cardiopulmonary insufficiency problems [4]. Therefore, it is valuable to repair rib defects by means of tissue-engineered bone materials. In order to evaluate and test the potential applications of all kinds of bone substitute materials in rib defect repair and inducing injured rib regeneration, an ideally canonical rib critical size defect (CSD) model should be established initially. However, the basic parameters like critical size of rib defect in animal model are short of reference in literature so far, although critical size in many other sites of bone defect models (rabbit radial [5, 6], minipig mandible [7, 8], rat calvarium [9] etc.) had been built well.

But till now, no literatures were reported on the determination of critical size for rabbit rib defects. Therefore, in this work, we developed a rabbit rib CSD model and determined the surgical procedure and critical size values, which will be able to provide a reference for the mechanism study on rabbit rib defect repair and future rib defect repair and the CSDs of human rib segmental defects.

The CSD is traditionally defined as the smallest size of the intraosseous wound in a particular bone and species of animal that will not heal spontaneously during the lifetime of the animals [10–12]. Practically, because it is extremely difficult to monitor the healing characteristics of a defect in an animal during its lifetime, recent studies redefined a CSD as one does not heal within the duration of the study [13]. In this study, it is also worthwhile to mention here that a rabbit rib defect repair is relatively fast, we used the investigation time is up to three months. Besides, it is interesting to note that many researchers have previously reported that the involvement of periosteum will significantly affect the critical size of bone tissues. Therefore, in the case of rabbit rib, two different CSDs about periosteum were considered.

## Experiments

### Ethics and materials

The animal experiments in this work were approved and performed in strict accordance with the regulations and guidelines by the Institutional Animal Care and Use Committee of Liaocheng People’s Hospital, P. R. China. All the surgeries were performed under general anesthesia, all the efforts were made to minimize animals suffering. Ten 4-month-old, clean grade, male, New Zealand white adult rabbits with an average weight of 3.0 kg (range, 2.5–3.5 kg) were purchased from the Jinfeng Feed Co., Jinan, P. R. China.

### Surgical procedure

**Figure 1.**
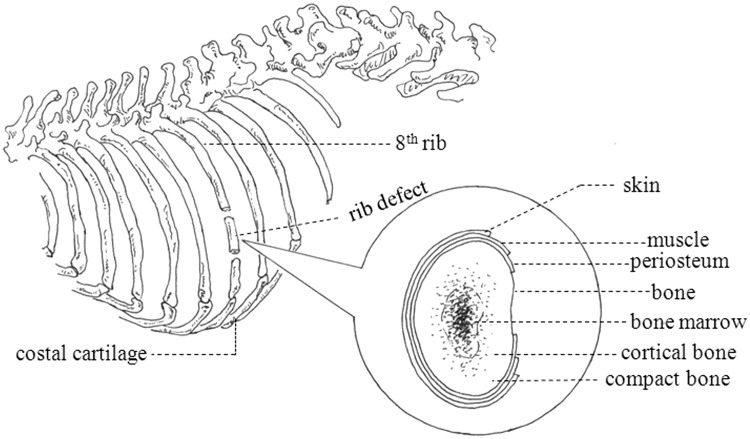
The diagram of rib defect position and periosteum structure

All animals were divided into two groups: group A (No.1-6 rabbits) with periosteum reserved and group B (No.7-10 rabbits) with periosteum removed (As shown in Table 1). The eighth rib of right abdomen was chosen for the experimental rib (as shown in Fig. 1) and the structure and location of periosteum are shown in appended figure of Figure 1.

**Table 1.** Defect size, location and status of rib

Rabbit number	Periosteum	Designed defect size (cm)	Defect location
1–2	Yes	3	Eighth rib
3–4	Yes	4	Eighth rib
5–6	Yes	5	Eighth rib
7–8	No	1	Eighth rib
9–10	No	2	Eighth rib

Numbers 1–6 were in group A with the Periosteum; numbers 7–10 were in group B with Periosteum.

**Figure 2.**
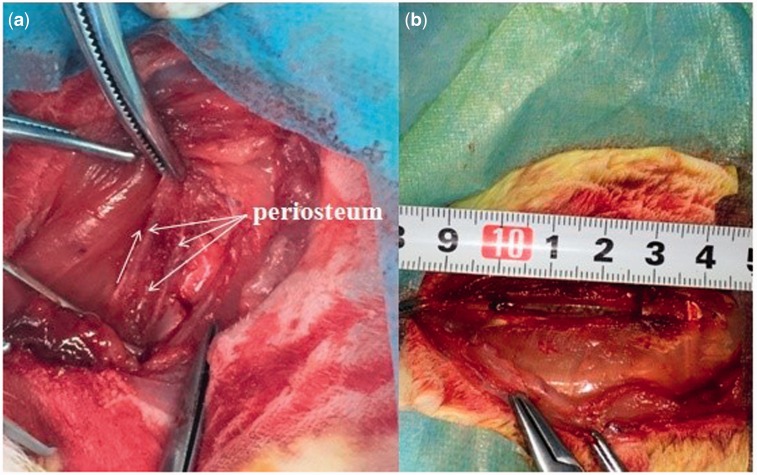
Experimental procedures. **(a)** group a: the periosteum was elevated and preserved (white arrows, periosteum on the both sides and bottom), **(b)** the rib bone segment (3 cm-defect) was removed

**Figure 3.**
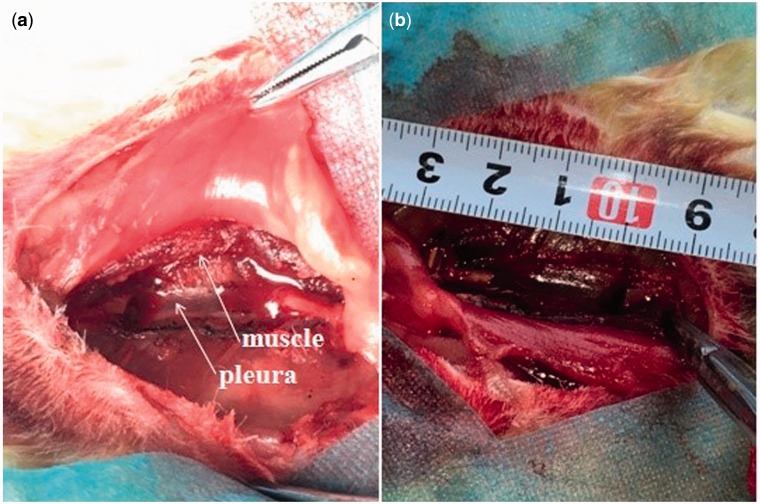
Experimental procedures. **(a)** group B: the periosteum was removed by electric bright technology (white arrow, pleura, muscle), **(b)** the rib bone segment (2 cm-defect) was removed

All the surgical procedures were performed under systemic anaesthesia using 10% (vol/vol) chloral hydrate in oxygen for ∼2 min. Assessment of the depth of anesthesia is according to the lack of reflex to toe pinch. After anesthetizing, the rabbits were immobilized in a lateral position. The right abdomen was upward and the surgical site was shaved, isolated with sterile drapes, disinfected with iodine and alcohol. A skin incision was made on the right flank, from which the skin, subcutaneous fascia and deep fascia were incised. An orthopedic ribs scissors was used to remove a section of the ribs (As shown in Fig. 2a). The edges of the rib defects were smoothed using a rasp to attain the designed size with the help of the caliper. In group A, the rib bone segments of 3–5 cm were removed and the periosteum on the both lateral margin and bottom was preserved (as shown in Fig. 2b). In group B, the rib bone segments of 1 and 2 cm were removed and all the periosteum was removed by electric bright technology (As shown in Fig. 3a), the pleura and muscle were appeared (as shown in Fig. 3a, white arrow). The bone segment (2 cm-defect) was removed (as shown in Fig. 3b). The rib bone fragments, related coagulation scab and rib bone marrow tissues were washed with 50 ml normal saline. After hemostasis and wound rewashing, the fascia, subcutaneous tissues and the skin incision were sutured with absorbable 4/0 surgical sutures by suturing in two layers after saline irrigation. The wound was disinfected with iodine and alcohol. After surgery, the rabbits were intramuscularly injected with penicillin every day for 3 days in succession. The rabbits were housed separately.

### Postoperative observation, X-ray and histological examinations

The experiment rabbits were kept in separate cages at room temperature (20–25°C) and exposed to the natural and artificial lighting with a 12/12-h light/dark cycle. The rabbits were fed with nutrient rabbit feed. The breathing, body temperature, incision infection and the rabbits movement function were investigated.

Ordinary X-radiograph examinations were underwent by the digital X-ray machine (46 kV, 6.0 mA, 10 ms, Siemens AG, Munich, Germany) at the 4th, 8th and 12th week postoperatively. The rabbits were anaesthetized and ketamine (40 mg kg^−1^) by intramuscular injection and lay on their left position. Then, photographic film was placed on the left abdomen and X-ray photos were underwent.

The experiment rabbits were sacrificed at the 12th-week postoperatively. The specimens from bone defect sites were fixed in 10% neutral buffered formalin for 48 h. Then they were fixed with 10% paraformaldehyde, and were decalcified by 5% ethylene diamine tetraacetic acid. After decalcification, the specimens were cut on the microtome, then embedded in lab-grade paraffin wax. The longitudinal plane sections were prepared and stained with hematoxylin and eosin (H&E). Then they were observed using a light microscope (IDA-2000, Konghai Technology and Development Co., Beijing, P. R. China).

## Anticipated results

### General observation

All experiment rabbits after surgery described above recovered quickly, returned to routine activities such as grooming, eating and drinking within 48 h. No apparent signs of infection were observed. The animals in group A gained more weight than those in group B during the experimental period.

### Radiology evaluation

**Figure 4.**
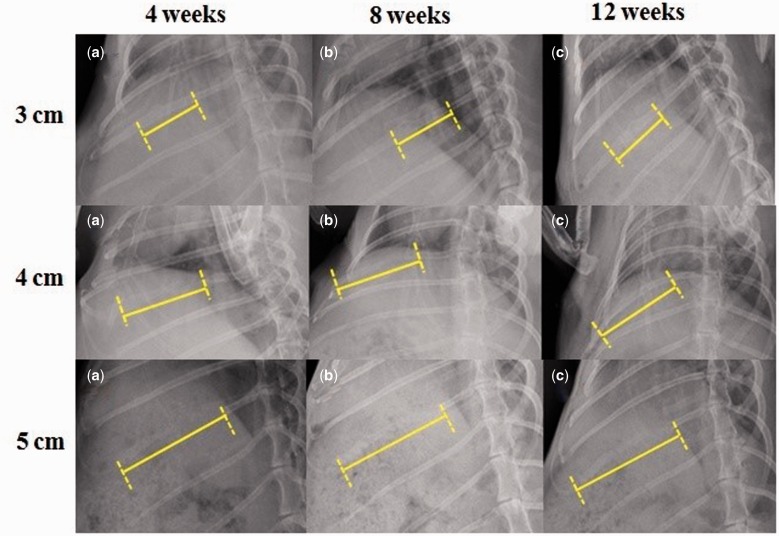
X-Ray radiographs of rabbit rib defects for group a (periosteum preserved). The X-rays at the 12th week postoperatively showed that 3- and 4 cm-defects were healed, but 5 cm-defects were not completely healed

**Figure 5.**
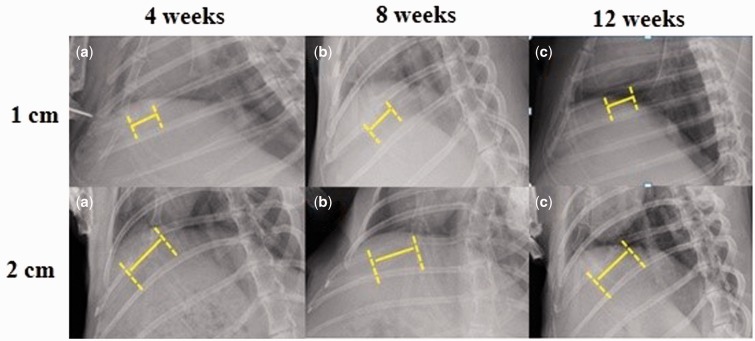
X-Ray radiographs of rabbit rib defects for group B (periosteum removed). The x-rays at 12 weeks after surgery showed that the 1 cm-defect had healed, but 2 cm-defect had not completely healed

X-ray radiographs showed the 3- and 4 cm-defects of group A were covered with pronounced opacity and new bone callus (as shown in Fig. 4) and the 1 cm-defect of group B (as shown in Fig. 5) at postoperative week 12. In contrast, X-ray radiographs showed the 5 cm-defect of group A was not healed completely with a remaining gap between the rib bone stumps (as shown in Fig. 4) and the 2 cm-defect of group B (as shown in Fig. 5). As shown in Figure 4, the 3- and 4 cm-defects showed partically regenerated at postoperative week 4. At 12 weeks after surgery, the rib defects were covered with newly-formed calluses and spontaneous healing and completely regenerated. The 5 cm-defect showed little bone formation at 4 weeks postoperatively, and the 5 cm-defect failed to heal at 12 weeks after surgery. In group B, obvious radiolucency was also observed in the 2 cm-defect (as shown in Fig. 5) at postoperative week 12. When compared with group A, new bone formed in group B with a significantly lower bridging rate and the gaps could still be seen at postoperative week 4. Furthermore, the 5 cm-defect of group A and 2 cm-defect of group B were underwent X-ray examinations at postoperative week 16, we found almost the same results as the 12^th^ week after surgery.

### Histological examinations

**Figure 6.**
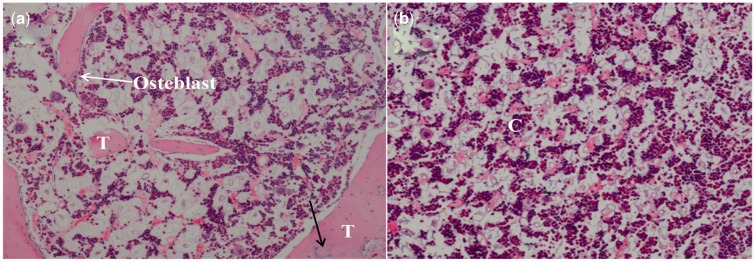
Hematoxylin-eosin photographs (H&E × 40 magnification) of rabbit rib defects at 12 weeks after surgery. **(a)** group a (periosteum preserved) showed intramembranous ossification, T: new bone trabecula. **(b)** group B (periosteum removed) showed cartilaginous ossification, C: chondrocytes

Histological examinations of 3 cm-rabbit rib defect in group A showed that new bone formed via intramembranous ossification, as shown in Figure 6. The H&E staining images displayed that osteoblasts could be detected in group A (Fig. 6a, white arrow), which showed this area should be the new bone tissue. The new trabecula bone formation that indicated the defects were repaired. The mature Haversian system was found (Fig. 6a, black arrow). However, chondrocytes were observed in group B (Fig. 6b), which meaned cartilaginous ossification occurred in group B. More new bone-like tissues was found in group A than group B. Previous studies demonstrated that intramembranous ossification was effective in terms of the osteogenesis rates and the new bone quality [7].

## Discussion

The healing of critical size bone defects is a key determinant in orthopedic surgery. The experimental animal’s age, weight, gender, and defect type all affected the rate of bone union [10]. For example, CSD in Wistar rats femur is 5 mm [11], in C57BL/7 mice femur is 2.5 mm [12], while, the CSD (diameter) in an adult guinea pig within the calvaria is 8 mm [13], and the CSD in dogs ulna is 2.0 cm [14]. The rabbit rib defect used in this study was younger and likely had a stronger regenerative potential than did the other defect type used in their study. It is also likely that the rib defect examined in our study could be easier to heal than the other defect examined in their study because of the total bone voxel count, such as the complex and continuity of bones surrounding environment and the abundant tissue and blood supply. Therefore, our CSD model can provide a reference for the CSDs of human rib segmental defects and be used to study the treatment modalities such as autologous rib-associated tissue engineering approaches.

Many experts had demonstrated the capacity of the periosteum for ossification many years ago. Some researchers had elucidated periosteum played a regenerative role in repairing large segmental defects. Geiger et al. [15] created a radial defect model by resecting defect segments including the periosteum in the distal radius. In this work, they found that when the periosteum was preserved, bone formation was promoted. However, when the periosteum was removed, cartilaginous ossification only depended on the bone tissues. Therefore, the CSD with periosteum was larger than that without periosteum under the same conditions. Periosteum is a specialized highly vascularized connective tissue, which envelopes rib bone surfaces (as shown in Fig. 1). It is composed of an external fibrous layer containing elastic fibres and microvessels and an inner cambium layer [16, 17]. The thin cambial layer contains most of the cells and the thicker fibrous layer can be divided into a matrix layer and a fibroblastic/collagenous layer aligned in the direction of bone growth, expands in this way with the growth of bone [18]. This demonstrated that the periosteum provides an intriguing niche for mesenchymal stem cells and a source for molecular factors that modulate the behavior of bone cells which has a large potential for ossification and act as major players in bone development, fracture healing and regenerated the injured bone repair [19]. But the muscle tissue adverses to the behavior of bone cells where the periosteum is lacking or damaged to cause developmental abnormalities. This stated that the development of substantial regenerated bone is unlikely in bones without periosteum. Furthermore, we can make a better understanding of the role of periosteum as the barrier membrane bounded all bones.

## Funding

Science Foundation of Shandong Province of China (ZR2015EL002) and National Natural Science Foundation of China (51572144).

## Conclusion

Overall, our results have demonstrated that the CSD of the rabbit rib was 5 cm when the periosteum preserved and 2 cm when the periosteum removed. This investigation of the CSD for the rabbit rib may provide a standard for the evaluation of bone biomaterials and the bone repair techniques.
